# The Impact of Minerals on Female Fertility: A Systematic Review

**DOI:** 10.3390/nu16234068

**Published:** 2024-11-27

**Authors:** Celine Kapper, Patrick Stelzl, Peter Oppelt, Clara Ganhör, Ayberk Alp Gyunesh, Barbara Arbeithuber, Marlene Rezk-Füreder

**Affiliations:** 1 Experimental Gynaecology, Obstetrics and Gynaecological Endocrinology, Johannes Kepler University Linz, Altenberger Strasse 69, 4040 Linz, Austria; peter.oppelt@kepleruniklinikum.at (P.O.); gyunesh.ayberk_alp@jku.at (A.A.G.); barbara.arbeithuber@jku.at (B.A.); marlene.rezk-fuereder@jku.at (M.R.-F.); 2 Department for Gynaecology, Obstetrics and Gynaecological Endocrinology, Kepler University Hospital, Johannes Kepler University Linz, 4020 Linz, Austria; patrick.stelzl@kepleruniklinikum.at; 3 Division of Pathophysiology, Institute of Physiology and Pathophysiology, Medical Faculty, Johannes Kepler University Linz, 4020 Linz, Austria; clara.ganhoer@jku.at; 4 Clinical Research Institute for Cardiovascular and Metabolic Diseases, Medical Faculty, Johannes Kepler University Linz, 4020 Linz, Austria

**Keywords:** female fertility, minerals, oocyte quality, miscarriage, hormonal regulation, IVF

## Abstract

Female fertility and reproductive system disorders are influenced by a complex interplay of biological, physiological, and environmental factors. Minerals have emerged as crucial yet often overlooked elements that impact fertility and the prevalence of reproductive system disorders. Background/Objectives: This review aims to provide a comprehensive overview of the multifaceted role of minerals in female fertility, focusing on key areas such as oocyte quality, ovulation, embryo development, oxidative stress, miscarriage, hormonal regulation, environmental exposure, and in-vitro fertilization (IVF) outcomes. Methods: A systematic review was conducted, focusing on randomized controlled trials (RCTs), prospective cohort studies, case-control studies, nested case-control, and observational studies examining mineral supplementation and nutrition in women planning pregnancy or utilizing assisted reproduction technologies (ARTs). Relevant literature was sourced from multiple electronic databases, including PubMed, Scopus, Google Scholar, Web of Science, and the Cochrane Library, using keywords related to minerals and female fertility. The quality of studies was assessed using the Newcastle–Ottawa Scale (NCO) for non-randomized studies and the Risk of Bias (RoB) tool for RCTs. This systematic review has been registered on PROSPERO (registration number is CDR 42024547656). Results: From an initial pool of 20,830 records, 39 articles met the inclusion criteria and were analyzed. The studies addressed various reproductive outcomes influenced by minerals: embryo development, oocyte quality, oxidative stress, miscarriage, hormonal regulation, IVF outcomes, environmental exposure, and minerals as biomarkers. The analysis revealed that minerals like selenium, zinc, and copper are essential for maintaining reproductive health, while exposure to toxic metals such as cadmium and lead is detrimental. Conclusions: This review highlights the crucial role of both mineral supplementation and serum mineral status in female fertility. The findings provide key insights for clinicians to improve reproductive health through targeted mineral intake and monitoring. Further research is needed to refine guidelines for supplementation and serum levels in women with fertility issues.

## 1. Introduction

Female reproductive health encompasses a range of issues, including infertility, which is defined as the inability to conceive after a year or more of regular unprotected intercourse and affects approximately 10–15% of couples worldwide [1,2]. The causes of infertility are diverse and often multifactorial, including ovulatory dysfunction, tubal or pelvic pathology, sperm abnormalities, as well as unexplained factors [1,2]. Lifestyle factors such as weight, stress, smoking, and alcohol consumption can also significantly influence conception [3,4]. This modern lifestyle, characterized by altered dietary habits, exposure to environmental pollutants, and increased stress, profoundly impacts an individual’s mineral status [5,6]. The exposure to heavy metals like lead and cadmium can antagonize mineral absorption and function, potentially worsening fertility challenges [7]. While the influence of minerals on male fertility has been intensively investigated [8,9,10,11], their role in women’s reproductive health has been marginally addressed, often only in the context of specific reproductive pathologies [12], emphasizing vitamins and multivitamin supplementation [13]. This review aims to address these gaps. We focus on minerals and their distinct roles in miscarriage, hormonal regulation, ovulation, oxidative stress, and oocyte quality. It provides a comprehensive overview of the role of minerals in female fertility and gives insights into potential therapeutic approaches for fertility challenges by offering evidence-based recommendations for mineral intake in reproductive-age women.

While essential minerals such as selenium, zinc, and copper play an important role in female reproductive health, cautious use of supplements is necessary. Without a confirmed deficiency or medical indication, mineral supplementation could potentially cause more harm than benefit. This review emphasizes that supplementation should be considered only when a deficiency has been identified.

## 2. Materials and Methods

### 2.1. Research Question

In this systematic review, we aimed to explore the impact of minerals on female fertility. We focused on randomized controlled trials (RCTs), prospective cohort studies, case control studies, nested case-control studies, and observational studies examining mineral supplementation and nutrition in women planning pregnancy or utilizing assisted reproduction technologies (ARTs).

### 2.2. Eligibility Criteria

Our inclusion criteria were focused on studies that specifically investigated the role of minerals in female fertility, were conducted on human subjects, and published in peer-reviewed journals in English. We excluded studies that did not directly relate to the impact of minerals, were animal studies or in vitro studies, and those that were reviews, editorials, or commentaries without primary data.

### 2.3. Study Selection

To compile a comprehensive list of relevant literature, we conducted searches across multiple electronic databases, including PubMed, Scopus, Google Scholar, Web of Science, and the Cochrane Library. The studies were categorized using the PECOS methodology [14]. (Population, Exposure, Comparison, Outcome, Study Design) to define key variables (Table 1). Our search strategy incorporated a combination of keywords and phrases such as “minerals”, “female fertility”, infertility”, “reproduction”, “recurrent pregnancy loss (RPL)”, “spontaneous abortion”, “minerals”, and “trace elements” and specific minerals, such as “iron”, “selenium”, “zinc”, “calcium”, “magnesium”, “lead”, and “copper”. Boolean operators (AND, OR) were utilized to refine the search parameters. The literature search was confined to articles published from January 2005 to November 2023.

### 2.4. Screening Process

To ensure the inclusion of high-quality studies, we employed a rigorous quality assessment using PRISMA (Preferred Reporting Items for Systematic Reviews and Meta-Analyses) guidelines [15]. This assessment covered various dimensions including study design, methodology, sample size, statistical robustness, and direct relevance to the research question. Studies that did not meet our predetermined quality threshold were excluded from the review.

Through this structured and systematic methodology, we aimed to provide a thorough and unbiased analysis of the current literature on the impact of minerals on female fertility and reproductive health disorders.

From an initial pool of 20,830 records, studies were screened and excluded based on predetermined criteria. Titles and abstracts were reviewed to remove non-clinical studies (e.g., in vitro, animal studies), secondary sources (e.g., reviews, commentaries), and studies without primary data. Full-text articles were then assessed for quality and relevance, with exclusions based on insufficient methodology, inadequate population focus, language, and accessibility issues. This rigorous selection process, detailed in the PRISMA flow diagram, ensured that only high-quality, clinically relevant studies were included in the final analysis, resulting in a selection of 39 studies.

### 2.5. Study Quality Assessment and Data Extraction

For the assessment of study quality and data extraction in our systematic review, we used the Newcastle–Ottawa Scale (NCO), a tool designed to evaluate the quality of non-randomized studies by assessing selection, comparability, and outcome criteria [16]. For randomized controlled trials, we used the Risk of Bias (RoB 2 tool), which systematically evaluates methodological rigor across domains like randomization and outcome reporting [17]. These tools enabled a systematic assessment of the methodological rigor of each study, with a particular focus on aspects such as the selection of study groups, the comparability of groups and the measurement of outcomes. Each study was assessed on the basis of specific criteria such as the clarity of the definition of selection criteria, the directness of exposure and the independence of outcome measurement. This thorough evaluation ensured that our review was based on reliable and high-quality evidence, which minimized the potential influence of bias on our findings.

## 3. Results

**Figure 1:**
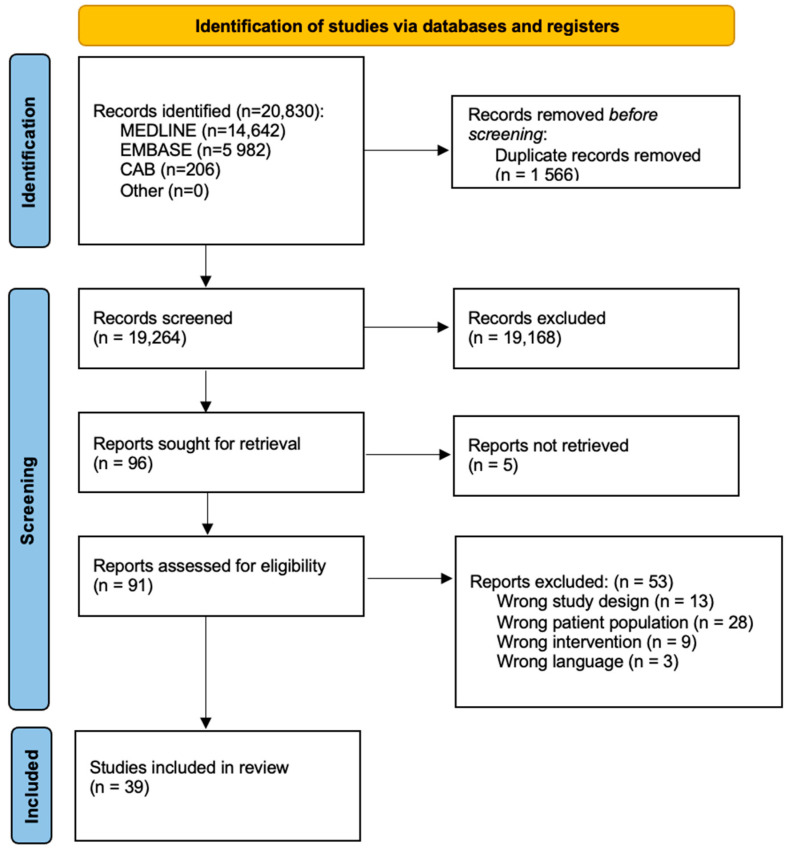
Flowchart of the process to select and include studies for a systematic review of the impact of minerals on female fertility.

From an initial pool of 20,830 records, we identified and removed 1566 duplicates. Subsequently, we screened 19,264 titles and abstracts, narrowing down the selection to 96 articles for in-depth full-text review (refer to Figure 1). Through this meticulous process, 39 articles ultimately satisfied the inclusion criteria and were selected for inclusion in our study.

The studies included comprised one double-blinded randomized controlled trial [18], 1 multicenter randomized prospective study [19], 1 randomized controlled trial [20], 1 two-center cross-sectional study [21], 2 cross-sectional studies [22,23], 1 observational study [24], 2 pilot studies [25,26], 16 case control studies [27,28,29,30,31,32,33,34,35,36,37,38,39,40,41,42] and 11 prospective cohort studies [43,44,45,46,47,48,49,50,51,52,53].

The systematic review was conducted following the guidelines of PRISMA (Figure 1) and has been registered on PROSPERO (registration number is CRD42024547656).

**Figure 2:**
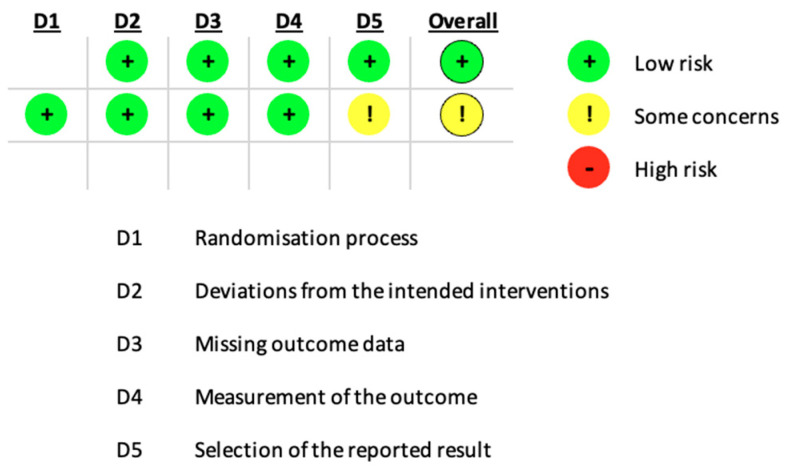
Detailed Risk of Bias (RoB) assessment for randomized controlled trials.

In our systematic review, an in-depth Cochrane Risk of Bias analysis was conducted for the included randomized controlled trials (RCTs). Each study was assessed across domains such as selection bias, performance bias, and detection bias, classified as ‘low,’ ‘some concerns,’ or ‘high’ risk based on specific criteria outlined in Figure 2. This approach ensures transparency in our methodological assessment and strengthens confidence in the overall reliability of included studies.

Figure 2 presents a detailed RoB analysis for two randomized controlled trials included in our systematic review. Each domain’s risk is marked with intuitive symbols: green plus signs indicate a low risk of bias, yellow exclamation marks represent some concerns, and the red minus sign points to a high risk. This detailed assessment of each domain—from the randomization process to the selection of reported outcomes—highlights specific areas where methodological rigor is well maintained and where caution may be warranted in the interpretation of study results. The red minus sign indicates a high risk of bias in the respective domain, which was not observed in our data. Overall, the studies are predominantly low risk in most areas, although there are some concerns about the measurement and reporting of outcomes, which warrants careful consideration of these results.

**Figure 3:**
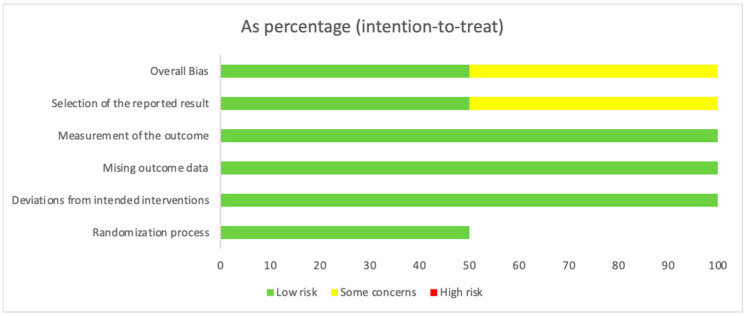
Aggregate Risk of Bias (RoB) as a percentage across bias domains.

Figure 3 depicts the aggregate bias risk as a percentage, illustrating the proportion of low, some concerns, and high risk across the five bias domains in an intention-to-treat analysis. The graphical representation through bar graphs allows for an immediate visual grasp of the potential impact that bias might have on the study’s evidence quality. The predominance of green in the bars suggests a low risk of bias, affirming the reliability of the evidence presented. However, the presence of yellow and red segments serves as a critical reminder to interpret specific aspects of the results with an appropriate degree of scrutiny.

### 3.1. Study Characteristics

**Figure 4:**
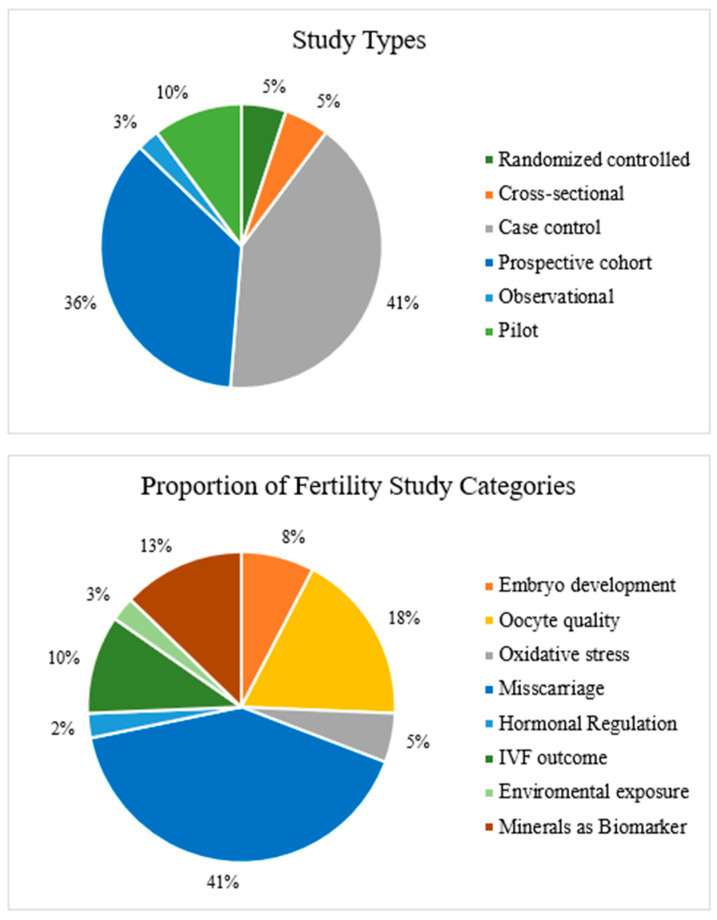
Frequency of study designs used (*n* = 39) and coefficients of fertility disorders included in the systematic review.

Among the 39 studies included in this review, a multifaceted approach was taken to evaluate the connection between mineral intake and female fertility outcomes. The studies are categorized as follows: embryo development (*n* = 3), oocyte quality (*n* = 7), oxidative stress (*n* = 2), miscarriage (*n* = 16), hormonal regulation (*n* = 1), IVF outcomes (*n* = 4), environmental exposure (*n* = 1), and minerals as biomarkers (*n* = 5), as seen in Figure 4. High risk refers to significant methodological concerns that may affect the reliability of the study’s findings, but no such concerns were identified in our data.

Table 2 synthesizes the results, organizing them by the specific reproductive outcomes they address, such as the implications of minerals on embryo growth, oocyte integrity, and the potential predictive value of mineral levels in IVF success rates. This tabulation aims to distill the complex interplay between dietary and environmental minerals on reproductive health, providing a streamlined reference for future research and clinical application.

The quality of the included studies was rigorously assessed using the Newcastle–Ottawa Scale (NCO). This tool evaluates non-randomized studies on group selection, comparability, and outcome assessment. Studies were rated as high, medium, or low quality based on a star system—reflecting low to high bias risk, respectively.

**Figure 5:**
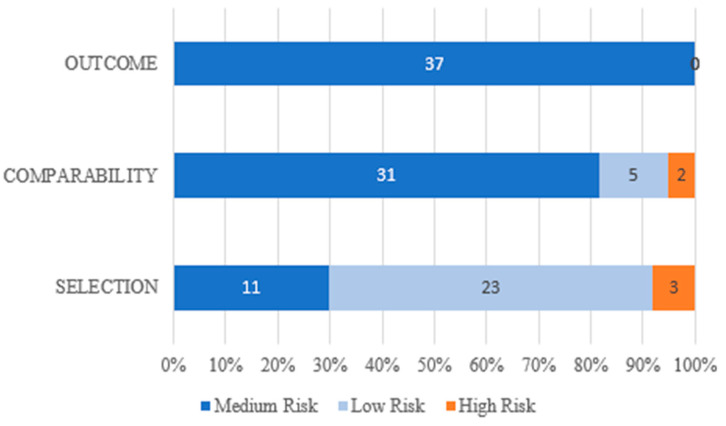
Quality assessment using the Newcastle–Ottawa Scale in the systematic review of the impact of minerals on female fertility.

Figure 5 presents the quality assessment of the included studies, focusing on the risk of bias across three critical domains: study selection, comparability, and outcome. For study selection, 23 studies were evaluated as low risk, while 11 studies were considered to have a medium risk, and 3 studies a high risk. In the domain of comparability, only 2 studies were identified as medium risk, with 31 studies at medium risk and 2 at high risk. Regarding the assessment of outcomes, no studies were categorized as having medium or high risk; instead, 37 studies were determined to be at low risk of bias. This breakdown reveals a spectrum of risk levels, with a particular need for scrutiny in the comparability domain.

**Figure 6:**
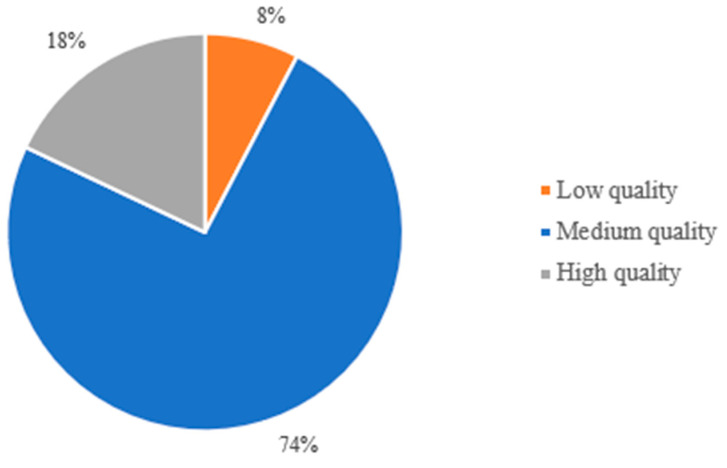
Distribution of risk of bias for all studies included in the systematic review.

Figure 6 uses the ROB and Newcastle–Ottawa scales to represent the quality of 39 studies. It finds that 3 studies are of low quality, meaning they may have significant issues. A total of 29 studies are of medium quality, indicating they are reasonably reliable but could have some biases. The remaining 7 studies are of high quality, showing they are well-conducted and likely to be very reliable. This helps us understand the overall trustworthiness of the research reviewed.

#### 3.1.1. Oocyte Quality and Ovulation

Seven studies evaluated the association between mineral intake and both oocyte quality and ovulation rates. This compilation included one double-blind randomized controlled trial [18], three prospective cohort studies [51,52,53], one case-control study [28], one two-center cross-sectional study [21], and one observational study [24].

##### Iron

A prospective cohort study from Massachusetts, involving 18,555 premenopausal women over eight years, led by Jorge E. Chavarro, revealed a significant link between iron supplementation and a lower risk of ovulatory infertility. The research found that women taking iron supplements had a notably reduced risk (40% lower) of developing infertility due to ovulatory issues compared to those not taking supplements. This finding underscores the potential of dietary and supplemental iron in enhancing fertility prospects among women of reproductive age [53].

In a study by Iris Holzer et al. in Austria, involving 72 participants, it was discovered that women with unexplained infertility often had ferritin levels below 30 µg/L. This case-control study, contrasting iron status between women with unexplained infertility and healthy controls, highlighted the importance of ferritin as a potential biomarker for infertility assessment. The findings suggest the necessity of including ferritin level screening in fertility evaluations, pointing towards its diagnostic value in identifying women at risk for unexplained infertility [28].

Lastly, an observational study from Massachusetts with 582 participants identified that high iron supplementation, specifically above 45 mg/day, is linked to decreased ovarian reserve, suggesting potential risks of excessive iron intake on fertility. This association raises questions about the potential gonadotoxic effects of excessive iron consumption, suggesting a delicate balance in iron supplementation. In studies conducted between 2007 and 2019, a significant decrease in the number of antral follicles and an increase in FSH levels on the third day were found in women with high iron consumption, suggesting that the level of iron supplementation in women undergoing fertility treatment needs to be carefully considered [24].

A two-center cross-sectional study [21] examined the iron status of Ghanaian nulliparous women and revealed a worrying prevalence of anemia and iron deficiency. Specifically, more than one-third of the participants had iron stores below the critical threshold, which is characterized by serum ferritin levels below 15 ng/dL. The analysis revealed that younger age, specific regional residence, and the presence of moderate anemia were the main factors for this nutrient deficiency. These findings underscore the urgent need for increased nutritional interventions and measures to improve iron levels in this population and highlight the importance of prenatal care in ensuring healthier pregnancy outcomes [57]. The study underscores the critical importance of iron for reproductive health, highlighting how iron deficiency, particularly among young pregnant women, represents not only a prevalent health issue but also potentially impacts oocyte quality and fertility.

##### Calcium

In a double-blind randomized controlled trial, the effect of calcium infusion on preventing ovarian hyperstimulation syndrome (OHSS) in 200 high-risk women undergoing IVF/ICSI was assessed. Participants were divided into two groups: the intervention group received calcium gluconate infusions, while the placebo group received saline solution, both administered on the day of ovum pick-up and the following three days. The results showed a significant reduction in the incidence of OHSS in the calcium group (7%) compared to the placebo group (23%), with no severe OHSS cases in the calcium group versus 4% in the placebo. The study concluded that calcium infusion is an effective preventative strategy for OHSS without impacting pregnancy rates [18]. While this study does not directly address fertility outcomes, it highlights the potential role of calcium as a preventive intervention in reproductive health management for women utilizing ART, minimizing the risk of OHSS—a significant complication associated with fertility treatments.

##### Iron, Calcium, and Sodium

A prospective study [51] examining the influence of follicular fluid metals on assisted reproduction outcomes, significant findings were highlighted regarding the relationship between trace metals and oocyte quantity and maturity. Specifically, a positive correlation was identified between iron levels and the number of oocytes, suggesting that higher iron concentrations are associated with an increased number of oocytes.

Conversely, calcium demonstrated a negative correlation, indicating that higher calcium levels were linked to a lower number of oocytes. Similarly, for oocyte maturity, iron again showed a positive correlation, with higher levels contributing to a greater number of mature oocytes. In contrast, calcium, and sodium levels negatively affected oocyte maturity, where elevated concentrations of these metals were associated with a reduced number of mature oocytes. This study underscores the pivotal roles of specific minerals in the reproductive process, where iron positively correlates with both the quantity and maturity of oocytes, suggesting its beneficial impact on fertility, while elevated levels of calcium and sodium are linked to decreased oocyte quantity and maturity, highlighting the complex interplay of trace elements in assisted reproductive outcomes [51].

##### Mercury, Zinc, and Selenium

A 2011 study investigated the impact of heavy metals, specifically mercury, zinc, and selenium, on IVF outcomes in 30 subfertile women. By analyzing hair samples, a method reflecting long-term exposure, researchers found that higher mercury levels were negatively associated with oocyte yield and follicle number, suggesting mercury’s potential role as an endocrine disruptor adversely affecting ovarian response. Conversely, higher zinc and selenium levels were positively associated with oocyte yield, with selenium also positively correlating with follicle number [52].

#### 3.1.2. Embryo Development

Three prospective cohort studies [51,54,55] investigated the effects of mineral intake on embryo development in ART treatments.

##### Copper, Selenium, and Zinc

A study focusing on intracytoplasmic sperm injection (ICSI) as part of IVF treatment [54]. Researchers evaluated the impact of trace metals cadmium (Cd), copper (Cu), iron (Fe), lead (Pb), selenium (Se), and zinc (Zn) in follicular fluid on IVF outcomes and embryo development and revealed that higher levels of Cu, Zn, and Se in the follicular fluid were associated with faster embryo development stages, while higher levels of Cd and Pb were linked to slower development [54].

##### Copper and Manganese

In a 2023 study in New Jersey [55], involving 60 IVF participants, researchers assessed the impact of eight essential trace elements on reproductive success by analyzing urine, plasma, and follicular fluid samples. The analysis revealed copper and manganese positively influenced ovarian response and embryo development, whereas higher lithium and molybdenum levels correlated with adverse outcomes like lower implantation and live birth rates. Additionally, elevated urinary lithium and chromium were linked to reduced chances of live birth [55].

##### Selenium

Furthermore, in a study by Yu Cao et al. [27], researchers explored the relationship between essential trace elements (copper, zinc, selenium, and cobalt) in peripheral blood and the risk of early embryonic arrest (EEA) among women undergoing IVF treatments. EEA refers to the situation in which an embryo fails to develop or divide in the early stages after fertilization, resulting in termination of the pregnancy before it can implant firmly in the uterus [58]. The findings revealed that selenium levels were significantly lower in the EEA case group, while cobalt levels were significantly higher, suggesting a possible link between these elements and the risk of EEA. No significant differences were observed in copper and zinc levels between the groups. The study suggests a potential link between trace element levels and EEA risk, highlighting the importance of selenium and cobalt in reproductive outcomes [27].

#### 3.1.3. Oxidative Stress and Fertility

Examining the impact of oxidative stress, this investigation includes a comparative analysis and a prospective cohort study, with a particular focus on zinc, copper, and manganese.

##### Selenium

The study by Singh et al. [50] examined oxidative stress and selenium levels in the follicular fluid of women undergoing IVF due to endometriosis or tubal infertility. The results showed that women with endometriosis had lower selenium levels and increased oxidative stress markers than women with tubal infertility. This selenium deficiency, an essential trace element known for its strong antioxidant properties, was particularly associated with poorer egg and embryo quality, which has a significant impact on IVF success rates. This suggests that selenium is critical in reducing oxidative stress to improve oocyte and embryo quality, highlighting the importance of maintaining adequate selenium levels to improve IVF outcomes. In addition, the study found that intrafollicular zinc levels were higher in women with endometriosis who successfully conceived after IVF, suggesting that in addition to selenium, zinc also plays a crucial role in promoting female fertility by potentially improving the environment for oocyte maturation and embryo development [50].

##### Zinc, Copper, and Manganese

The comparative study assessed [56] superoxide dismutase 1 (SOD1) and SOD2 activities, superoxide anion (SOA) levels, and the micronutrients zinc, copper, and manganese in non-pregnant women, healthy pregnant women, and recurrent miscarriage (RM) patients. SOD activities and zinc, copper, and manganese levels were significantly reduced in RM patients compared to healthy pregnant and non-pregnant women, with a concurrent increase in SOA levels across all samples, indicating oxidative stress. The findings suggest oxidative stress in RM patients’ blood and placental tissues as a potential cause of miscarriage, implying dietary supplementation of zinc, copper, and manganese might be beneficial pre- and post-conception for these patients [56].

#### 3.1.4. Miscarriage

In the investigation of miscarriage, including spontaneous abortion (SA) and recurrent pregnancy loss (RPL), a total of 16 studies were selected for inclusion. This comprised ten case-control studies, three prospective controlled trials, two cross-sectional studies, and one pilot study.

##### Copper, Zinc, and Selenium

Four studies assessed the relationship between copper, zinc, and selenium and miscarriage. In a 2023 prospective cohort study, researchers aimed to evaluate the association between serum markers (inflammatory cytokines and trace elements) and the risk of miscarriage among women undergoing IVF treatments. The study involved 200 women and measured the levels of essential trace elements (vanadium (V), copper (Cu), zinc (Zn), selenium (Se), and molybdenum (Mo)) and inflammatory cytokines (IL-1β, IL-6, IL-8, IL-10, and TNF-α) 14 days after embryo transfer. Results showed that miscarriage cases had significantly lower levels of IL-1β, TNF-α, V, Cu, Zn, and Se compared to successful live birth cases. Specifically, higher serum levels of Cu, Zn, and Se were associated with a lower risk of miscarriage, suggesting these trace elements as potential indicators of positive IVF outcomes [49].

A case-control study [29] evaluated antioxidant status and trace element levels (Se, Zn, Cu, Mn) in 83 women with miscarriages compared to 70 pregnant/postpartum women. Results showed lower total antioxidant status and serum copper, higher manganese in miscarriage cases, and altered trace elements in placental tissue. These findings suggest that antioxidant imbalance and trace element discrepancies may play a role in miscarriage occurrence [29]. However, it should be noted that this study [29] compares trace element levels in women at different reproductive stages (pregnancy, miscarriage, and postpartum), which may introduce metabolic variations that affect mineral levels independently of reproductive outcomes.

Another case-control study [30] found that pregnant women with a history of recurrent spontaneous abortions had significantly lower levels of zinc, copper, and vitamin E, and higher levels of selenium, lead, and cadmium compared to those without such a history. Additionally, there was a slight but not statistically significant decrease in progesterone levels in women with recurrent abortions [30]. Equivalent results were obtained in a study by Popović et al. [31] which included 35 cases of complete spontaneous abortions, 40 cases of miscarriages and 50 healthy pregnancies. The group of healthy pregnant women had higher average plasma copper concentrations and antioxidant enzyme activities (glutathione peroxidase and catalase) than the groups of spontaneous abortions and miscarriages [31].

##### Lead

The findings in relation to lead are also highlighted in a case-control study conducted at Peking Union Medical College Hospital between 2016 and 2018 [32], which examined the effects of lead levels on spontaneous abortions within 12 weeks of gestation by comparing 150 cases of spontaneous abortions with 150 control subjects with viable pregnancies. The results showed significantly higher mean lead levels in the case group (27.17 μg/L) compared to the control group (17.28 μg/L, *p* = 0.000). A higher risk of spontaneous abortion was associated with increasing lead levels, especially when lead levels exceeded 10 μg/L, emphasizing the significant impact of lead exposure on early pregnancy loss [32].

Although research [33] assessed the relationship between lower levels of prenatal toxic metal exposure and SA risk among pregnant women in Tehran, Iran. It compared blood metal concentrations between women who had SAs and those with live births. Results indicated higher, but not statistically significant, levels of lead, antimony, and nickel in the SA group. Logistic regression showed a significant association between maternal age and SA risk, with antimony positively related to SA risk (OR: 1.65). The study suggests minimizing exposure to these metals early in pregnancy to potentially reduce adverse outcomes.

##### Selenium

Five studies that assessed selenium and miscarriage were identified. A case-control study [34] investigated the relationship between maternal hair selenium levels and RPL, comparing women with RPL to those with successful reproductive histories. Despite significant socioeconomic and dietary differences between the groups, selenium levels in their hair were comparable and generally low. This finding is significant, considering selenium’s crucial role in reproductive health. The research did not establish a direct correlation between selenium deficiency and RPL but underscored the prevalent low selenium status among the participants [34]. Similarly, in research conducted in Warsaw, Poland [48], with a cohort of 74 expectant mothers, it was discovered that Se levels decreased significantly throughout pregnancy among both healthy individuals and those with autoimmune thyroid disease (AITD). This reduction in Se levels was evident across all trimesters, showing a significant portion of participants from both categories dropping below the World Health Organization’s definition of selenium deficiency (<45 μg/L).

These observations underscore the findings of Rayman et al. [47] who emphasize that selenium supplementation should be considered during pregnancy to prevent adverse outcomes for both pregnant women. The comparative study, which included 1197 women with singleton pregnancies, found that low maternal selenium levels at 12 weeks’ gestation were significantly associated with an increased risk of preterm birth. Specifically, women in the lowest quartile of serum selenium levels had a two-fold increased risk of preterm birth compared to women in the top three quartiles, even after accounting for pre-eclampsia. This suggests that low selenium status in early pregnancy may contribute to the occurrence of preterm births, including those due to premature rupture of membranes. The study highlights the potential importance of selenium in reducing inflammation and its possible role in preventing preterm birth and associated complications [47].

Finally, a case-control study [35] and pilot study [25] demonstrated that low serum selenium concentrations are associated with an increased rate of miscarriage and RPL. Abdulah et al. [35] showed that women who experienced miscarriages had significantly lower selenium concentrations, averaging 66.71 ± 13.55 ng/mL, compared to women with normal pregnancies, where the concentration was 76.36 ± 18.22 ng/mL. The activity of glutathione peroxidase (GPx), a selenium-dependent enzyme, was similar in both groups. The analysis suggests that selenium, regardless of GPx activity or smoking status, plays a crucial role in pregnancy maintenance. Supplementary, a pilot study from India [25] investigated selenium levels in red cells of 20 women with three or more unexplained recurrent pregnancy losses and compared them to a control group. Red cell selenium levels are considered better indicators of selenium status. The study found significantly lower selenium levels in the RPL group, with a mean of 119.55 ± 32.94 ng/mL, compared to the control group’s mean of 150.85 ± 37.63 ng/mL. The difference was statistically significant, suggesting selenium deficiency might be a risk factor for RPL.

##### Multiminerals

Five studies investigating the effects of multiminerals on miscarriage risk have been selected for this analysis. Research [36] assessing serum copper, iron, and manganese levels across healthy non-pregnant women, pregnant women, women who experienced miscarriages and women facing primary infertility revealed elevated Cu and Mn levels in pregnant subjects relative to controls. However, notable reductions in Cu levels were observed in those with miscarriage and infertility issues.

Building upon these insights, another study focused on the Persian population [37] examined blood levels of essential and non-essential metals in women with and without a history of spontaneous abortion. Results indicated lower levels of zinc and selenium and higher levels of Lead and arsenic (As) in women who experienced spontaneous abortions compared to controls. Elevated As and Pb were significantly associated with increased abortion risk, suggesting that reducing exposure to non-essential metals during pregnancy could decrease the likelihood of spontaneous abortion.

Furthermore, a cross-sectional study [22] explored the relationship between multiple trace elements on miscarriage risk during early pregnancy, analyzing pregnant women’s blood for element levels. Findings indicated that higher barium levels significantly increased miscarriage risk, whereas elevated levels of essential elements, such as copper and rubidium, were associated with reduced risk [22].

Another study [23] found that spontaneous abortion in the first trimester is associated with higher levels of non-essential metals (like arsenic, antimony, and bismuth) and disrupted metabolism of essential metals (such as magnesium, copper, and strontium). Additionally, decreased levels of key pregnancy hormones (estradiol and progesterone) and slight increases in lactate dehydrogenase and thyroid-stimulating hormone were observed among those who had a spontaneous abortion. Comprehensive analyses suggested that these hormonal imbalances and metal exposures are closely linked, underscoring the complex interplay between non-essential metal exposure, essential metal metabolism, and hormonal levels in affecting pregnancy outcomes.

In contrast, a study [38] involving 351 pregnant women aged 16 to 35 years investigated the relationship between moderate- to low-level lead exposure and the risk of spontaneous abortion. Blood samples were collected in the first trimester for lead measurement. The study found that mean blood lead levels did not significantly differ between those who experienced spontaneous abortions after the 12th week and before the 20th week of gestation and those with ongoing pregnancies, suggesting that low blood lead levels (mean < 5 μg/dL) in early pregnancy may not be a risk factor for spontaneous abortion in apparently healthy women [38].

#### 3.1.5. Hormonal Regulation

Apart from the study mentioned above [23], only one prospective cohort study has been conducted on the direct relationship between minerals and hormone regulation.

##### Sodium and Manganese

A study conducted by Kim et al. explored the association between dietary mineral intake and ovulatory function in 259 healthy, regularly menstruating women. This research aimed to determine how the intake of ten selected minerals influenced reproductive hormones and the risk of anovulation. Findings highlighted that low sodium intake (<1500 mg) compared to higher intake (≥1500 mg) was associated with increased levels of FSH and LH hormones and decreased progesterone levels. Additionally, low intake of sodium and manganese was linked to an elevated risk of anovulation. Other examined dietary minerals did not show significant effects on ovulatory function [46].

#### 3.1.6. Environmental Exposure

##### Selenium and Methylmercury

While Kim et al. spotlight the direct effects of nutrient intake on hormonal imbalances and anovulation, Maeda et al.’s [39] study expands this perspective by considering environmental exposures, particularly the harmful impact of methylmercury versus the protective function of selenium. In this case-control, involving 98 infertile women and 43 controls, researchers investigated how exposure to metals like methylmercury and selenium affects female fertility. Results showed infertile women had lower blood selenium levels and selenium/mercury ratios (189 ± 25 μg/L and 94.6 ± 44.3) compared to controls (200 ± 25 μg/L and 118.4 ± 70.5), highlighting selenium’s protective role against infertility. Conversely, after adjusting for age and selenium levels, infertile women exhibited higher mercury levels [39]. This link between dietary and environmental factors highlights the complexity of influences on female fertility, emphasizing the need for a holistic approach to assess and enhance reproductive health.

#### 3.1.7. IVF Outcome

Four studies assessed the IVF outcome and minerals. One multicenter randomized prospective study, two prospective cohort studies, a controlled clinical trial and one pilot study were examined.

##### Zinc

A 2021 study involving 305 women in China linked low serum zinc levels to higher In-Vitro-Fertilisation Embryotransfer (IVF-ET) failure rates in Shandong but not in Beijing, indicating regional dietary impacts on fertility. Specifically, lower zinc levels increased IVF failure risk by 66% in Shandong. The research suggests that adjusting dietary zinc intake could enhance IVF outcomes, especially in zinc-deficient areas. [45].

##### Magnesium and Calcium

A multicenter, randomized prospective study [19] examined the relationship between serum folate and total calcium and magnesium levels before ovarian stimulation and the outcomes of assisted reproductive technology (ART) in normogonadotropic women. Main outcome measures included total oocyte yield, mature oocytes, fertilization rate, biochemical pregnancy, clinical pregnancy, and live birth rates. Key findings included that higher serum folate levels (≥33.0 ng/mL) were associated with a significantly lower total number of oocytes retrieved and decreased odds of clinical pregnancy and live birth compared to women with lower folate levels (<10.8 ng/mL). Women with a higher Ca/Mg ratio (≥5.02) had significantly increased odds of biochemical pregnancy, clinical pregnancy, and live birth compared to those with a lower Ca/Mg ratio (<4.55). The study suggests that elevated baseline serum folate levels and a lower Ca/Mg ratio may be linked to poorer ART outcomes in normogonadotropic women [19]. In the study conducted by Grossi et al. [44] examining the effects of reproductive assistance on serum levels of magnesium and calcium, it was found that these essential minerals remained unchanged in infertile women undergoing intrauterine insemination (IUI) and IVF, despite the hormonal changes associated with ovarian hyperstimulation. However, a slight downward trend was observed, correlating with increased estrogen levels and Mg and Ca levels during IVF stimulation.

##### Multiminerals

In a 2017 study by Mary E Ingle et al., associations between IVF outcomes and essential trace elements, specifically cobalt, chromium, copper, manganese, molybdenum, and zinc, measured in follicular fluid and urine were investigated among 58 women. The study found positive associations between higher urine concentrations of cobalt, chromium, copper, and molybdenum and the number of oocytes retrieved, as well as the total number of embryos generated. Conversely, FF concentrations of chromium and manganese negatively impacted mature oocyte proportion, while FF zinc was inversely related to oocyte fertilization rates. Interestingly, no significant associations were observed between these trace elements and clinical outcomes such as implantation, pregnancy, or live birth [26].

In a controlled clinical trial [20], the effects of multivitamin/mineral supplementation on trace element levels in the serum and follicular fluid of women undergoing IVF were examined. Findings revealed that selenium and zinc levels in both serum and follicular fluid, and copper levels in follicular fluid, were lower in the IVF group compared to controls. Additionally, aluminum and iron levels were higher in the follicular fluid of IVF patients than in controls. However, the group receiving multivitamin/mineral supplements showed increased levels of aluminum, copper, zinc, and selenium in both serum and follicular fluid, and magnesium in serum, compared to the IVF group without supplements. Conversely, follicular fluid iron levels were lower in the multivitamin/mineral group than in the IVF group. The study concluded that multivitamin/mineral supplementation effectively normalized trace element levels in women undergoing IVF. The administration of a daily multivitamin/mineral supplement for 45 days to women undergoing IVF appeared to rectify these imbalances, normalizing the levels of copper, zinc, selenium, and magnesium in serum and follicular fluid, and reducing the elevated levels of iron in follicular fluid. This normalization suggests that supplementation could help in creating a more favorable biochemical environment for oocyte development and maturation, potentially improving the outcomes of IVF treatments [20].

#### 3.1.8. Minerals as Biomarkers

The selection comprises one randomized controlled trial, one prospective cohort study, and three case-control studies, emphasizing the significance of minerals as biomarkers.

##### Copper, Selenium, and Zinc

Additionally, Butts et al. [43] investigated the variability of essential and non-essential trace elements as biomarkers in the follicular fluid of 34 women undergoing IVF, to evaluate their influence on reproductive function. It revealed that inter-individual differences accounted for the majority of variability in FF trace element concentrations. Factors including age, BMI, race, smoking habits, infertility diagnosis, and IVF treatment protocol affected the variability of specific trace elements. The analysis showed that assessing 4–5 follicles suffices to estimate accurate subject-specific mean concentrations for biomarkers like Cu, Se, and Zn, while more than 14 follicles are necessary for reliable estimates of As, Hg, Cd, Pb, and Mn. This suggests that FF serves as a valid source of biomarkers for As and Hg exposure, guiding future research on trace element exposure in ovarian follicles and its impact on IVF success [43].

##### Cadmium and Chromium

In a study from 2016 to 2018 [40], involving 195 pregnant women, significant associations between toxic metal exposure and early pregnancy loss were identified. The participants were divided into two groups, 95 with spontaneous abortions within 12 weeks of gestation and 100 opting for induced abortions with observable fetal cardiac activity. Key findings showed that higher blood cadmium (>0.4 µg/L) and urine chromium (>2 µg/L) levels significantly correlated with increased odds of spontaneous abortion, highlighting these metals as potential biomarkers for embryotoxicity risk in the general population [40].

##### Copper, Iron, Zinc, Calcium, Magnesium, and Arsenic

A case-control study conducted a comparative analysis on the trace element content in hair between women with IVF-induced pregnancies and those with natural pregnancies, utilizing inductively coupled plasma mass spectrometry. It found that women undergoing IVF showed significantly lower levels of essential minerals such as copper, iron, zinc, calcium, and magnesium, and higher arsenic levels, compared to controls. These discrepancies highlight the need for regular monitoring and potential nutritional interventions to address these imbalances and support reproductive health in IVF-treated women [28,57].

##### Cadmium and Lead

Additional Omeljaniuk et al. [41] evaluated cadmium and lead levels in the blood and placental tissues of 83 women who experienced miscarriages, compared to 35 controls. Elevated Cd and Pb were observed in the miscarriage group, with smoking exacerbating Cd levels. The data suggest a correlation between higher Cd and Pb exposure and increased miscarriage risk, recommending monitoring these metals in prospective mothers. It proposes evaluating the ratio of toxic metals to antioxidants as a diagnostic marker to prevent miscarriages [41].

##### Copper, Magnesium, Iron, and Zinc

A 2023 nested case-control study [42] investigated the levels of serum zinc, copper, magnesium, and iron in pregnant women, comparing those with spontaneous abortions (SA, *n* = 80) at 5–12 weeks of pregnancy to controls without SA (*n* = 100). The findings revealed that maternal serum levels of Cu, Mg, Fe, and Zn were significantly lower in the SA group compared to controls (*p* < 0.005). The study suggests that measuring these minerals in early pregnancy could potentially predict the risk of SA, emphasizing the need to optimize micronutrient supplementation during pregnancy to prevent adverse outcomes [42].

#### 3.1.9. Dose–Response Relationships of Minerals in Female Fertility

Minerals play a central role in female fertility and influence factors such as egg quality, ovulation and pregnancy outcomes, as shown in the results above. Clinical studies show that both sufficient intake and optimal serum concentrations of these minerals are crucial to achieve positive effects on reproductive health. Conversely, deficiencies or an oversupply can have negative consequences.

The following tables provide an overview of the recommended daily doses (Table 3), the optimal serum levels (Table 4) and the clinical effects of under- or overdosing these essential minerals (Table 5). The information on recommended daily allowances (Table 3) comes from the World Health Organization (WHO) [59], the Food and Nutrition Board of the Institute of Medicine (IOM) [60,61], and the National Institutes of Health (NIH) [62].

The evidence shows that excessive amounts of individual minerals such as iron and copper are associated with health risks, which is contrary to a universal application.

## 4. Discussion

This review provides the most up to date and comprehensive summary of the literature on minerals and female fertility. We have delineated several minerals that correlate with enhanced fertility outcomes in women, which includes both the general population and those utilizing assisted reproductive technology.

### 4.1. Oocyte Quality

Iron supplementation is consistently linked to improved fertility outcomes [24,28,53], with studies showing a reduced risk of ovulatory infertility and emphasizing the potential of iron in enhancing fertility [24]. However, there is a caution against excessive intake, which could lead to decreased ovarian reserve, indicating the need for a balanced approach to iron supplementation. The positive impact of calcium, particularly through calcium infusion [18], in significantly reducing the incidence of ovarian hyperstimulation syndrome without affecting pregnancy rates, presents calcium as an effective intervention in assisted reproductive technologies. The relationship between minerals, especially iron, calcium, and sodium, with oocyte quantity and maturity reveals a complex interplay. Iron positively correlates with both the number and maturity of oocytes, suggesting its beneficial impact. In contrast, high levels of calcium and sodium are associated with reduced oocyte quantity and maturity, highlighting the delicate balance required in mineral intake for optimal reproductive outcomes [51]. The adverse effects of mercury contrasted with the positive impacts of zinc and selenium on IVF outcomes emphasize the need for awareness and management of heavy metal exposure in reproductive health [52].

### 4.2. Embryo Development

The summarized findings from three prospective cohort studies on the effects of mineral intake on embryo development in ART treatments reveal both commonalities and differences in how certain minerals influence reproductive outcomes [51,54,55]. Copper, Selenium, and Zinc were found to play pivotal roles in embryo development, with higher levels of copper, selenium, and zinc in follicular fluid associated with faster embryo development stages [54]. This suggests their beneficial impact on embryo growth and highlights the importance of a balanced trace mineral environment for successful IVF outcomes. The study from New Jersey [55] highlighted the positive influence of copper and manganese on ovarian response and embryo development. However, it also noted that elevated levels of lithium and molybdenum, as well as urinary lithium and chromium, were linked to adverse reproductive outcomes, such as lower implantation and live birth rates. This underscores the complex interaction between various trace elements and their collective impact on fertility success. Lower selenium levels were significantly associated with early embryonic arrest, suggesting selenium’s crucial role in early embryo development [27]. Conversely, higher cobalt levels were linked to increased EEA risk, indicating that not all trace elements support positive reproductive outcomes.

Across the studies, selenium consistently emerges as a critical element for embryo development, with its deficiency linked to poorer outcomes. Copper and zinc also appear beneficial for reproductive success. However, the studies also highlight the negative impact of certain elements, such as cadmium, lead, and potentially cobalt, which are associated with slower embryo development or increased risk of EEA.

### 4.3. Oxidative Stress and Fertility

Selenium levels were found to be lower in women with endometriosis undergoing IVF, linking selenium deficiency to increased oxidative stress and poorer egg and embryo quality [50]. This underscores the importance of selenium in reducing oxidative stress to improve oocyte and embryo health. In addition, higher intrafollicular zinc levels in women with endometriosis who became pregnant after IVF indicate the important role of zinc in promoting fertility. Reduced levels of zinc, copper, and manganese and increased oxidative stress markers in recurrent miscarriage patients compared to healthy pregnant and non-pregnant women indicate that oxidative stress may contribute to miscarriage [56]. This suggests that supplementation with zinc, copper, and manganese could be beneficial for women pre- and post-conception, particularly those at risk of recurrent miscarriages.

### 4.4. Miscarriage

Copper, zinc, and selenium are consistently highlighted across studies for their association with lower miscarriage risks [29,30,31,49]. Notably, higher serum levels of copper, zinc, and selenium are linked to positive IVF outcomes [49] and reduced miscarriage occurrences [31], underlining these minerals as potential indicators for reproductive success. Studies reveal that elevated lead levels are significantly associated with an increased risk of spontaneous abortion [32,33]. This emphasizes the adverse impact of lead exposure on early pregnancy loss, underscoring the importance of minimizing such exposure during pregnancy. While certain minerals like copper and manganese [36] are elevated in pregnant individuals, deficiencies in zinc and selenium [37], as well as elevated levels of non-essential metals such as lead [38] and arsenic [23], are linked to increased miscarriage risk. The collective findings from studies investigating the effects of multiple minerals on miscarriage risk suggest a delicate balance. While certain minerals like copper and manganese are elevated in pregnant individuals, deficiencies in zinc and selenium, as well as elevated levels of non-essential metals such as lead and arsenic, are linked to increased miscarriage risk. This indicates the complex relationships between essential and non-essential metal levels in pregnancy outcomes.

### 4.5. Hormonal Regulation

Adequate sodium and manganese intake is crucial for maintaining hormonal balance and ovulatory function. The study by Kim et al. [46] reveals that low sodium intake is associated with hormonal imbalances, specifically increased FSH and LH levels and decreased progesterone, which are linked to a higher risk of anovulation.

The research by Maeda et al. [39] highlights the contrasting effects of selenium and methylmercury on fertility, demonstrating selenium’s protective role against infertility and the detrimental impact of methylmercury. This underscores the importance of environmental factors in reproductive health and the protective benefits of certain minerals.

### 4.6. IVF Outcome

Low serum zinc levels were found to increase IVF failure rates, pointing to the critical role of zinc in successful IVF outcomes and the potential benefits of adjusting dietary zinc intake [45]. A study [19] examining the relationship between serum folate, calcium, and magnesium levels before ovarian stimulation found that a higher Ca/Mg ratio was associated with better ART outcomes, suggesting the importance of these minerals in enhancing reproductive success [44]. Research by Mary E Ingle et al. [26] showed positive associations between higher urine concentrations of cobalt, chromium, copper, and molybdenum with the number of oocytes retrieved and embryos generated. However, follicular fluid concentrations of some minerals negatively impacted oocyte quality, indicating the complex roles these elements play in fertility.

### 4.7. Minerals as a Biomarker

The comprehensive investigation into the role of minerals as biomarkers in the context of in vitro fertilization (IVF) and early pregnancy outcomes highlights the intricate relationship between trace element levels and reproductive health.

Individuals undergoing IVF displayed lower levels of critical minerals, specifically selenium, zinc, and copper, in their serum and follicular fluid, compared to control groups [43]. This discrepancy suggests a potential deficiency in essential nutrients crucial for successful fertility treatments. Conversely, these same individuals had elevated levels of aluminum and iron within their follicular fluid, indicating not only an imbalance in essential minerals but also exposure to potentially harmful elements [43]. The link between increased levels of cadmium (in blood) and chromium (in urine) with higher rates of spontaneous abortion further emphasizes the role of certain toxic metals as negative biomarkers for pregnancy outcomes [40]. These findings highlight the potential risk these metals pose to embryonic development and suggest their monitoring as part of prenatal care. Comparative analysis using inductively coupled plasma mass spectrometry on hair samples from women with IVF-induced pregnancies versus those with natural pregnancies revealed significant differences. Women undergoing IVF showed notably lower levels of vital minerals such as copper, iron, zinc, calcium, and magnesium [42], alongside higher levels of arsenic, underscoring the impact of mineral imbalances on fertility treatments and pregnancy health [28,57]. Additionally, evaluations of cadmium and lead levels in blood and placental tissues from women who experienced miscarriages compared to controls reinforced the adverse effects of these toxic metals on early pregnancy [41]. The association of lower maternal serum levels of copper, magnesium, iron, and zinc with an increased risk of spontaneous abortion suggests these minerals’ crucial roles in maintaining pregnancy.

The use of mineral supplements should follow a comprehensive clinical assessment and only be recommended when a deficiency is confirmed. This review highlights the significance of certain minerals, yet supplements are often taken without diagnostic evidence, which may pose more risks than benefits. For instance, excessive iron intake is associated with reduced ovarian reserve and other adverse effects. Therefore, recommendations to supplement selenium, zinc, and copper should not be generalized but rather tailored to specific clinical indications and individualized diagnostic evaluations to optimally support the health and well-being of patients.

## 5. Conclusions

In conclusion, based on this comprehensive review on the role of minerals in female fertility, it has become abundantly clear that each mineral examined—zinc, magnesium, calcium, iodine, selenium, iron, copper, and manganese—plays a significant role in the biological processes that underpin reproductive health. The interactions of these minerals with key determinants of fertility such as hormonal regulation, ovarian function, ovulation, oxidative stress, and embryo development are complex and critical (Table 2).

The evidence presented in this review illuminates the significant effect that both deficiencies and excesses of these minerals can have on fertility. This intricate balance of mineral levels underscores the importance of their careful management in dietary intake and potential therapeutic interventions.

## Figures and Tables

**Table 1:** PECOS characteristics and keywords in the search strategy for minerals and female fertility.

PECOS Guideline	Definition	Key Words Search
Population	Woman of reproductive age	Fertile, infertile, menstruating, women
Exposure	Mineral supplementation or deficiency	Mineral intake, deficiency, supplement, micronutrients, trace elements
Comparison	No supplementation or different levels of minerals	Placebo, no supplement
Outcome	Reproductive outcomes including fertility, miscarriage, and oocyte quality.	Fertility, pregnancy, oocyte quality, miscarriage, IVF outcomes, hormonal regulation
Study design	Randomized controlled trials, prospective cohort studies, case-control studies, observational studies	RCT, cohort, case-control, observational, clinical trial

**Table 2:** Impact of minerals on female fertility.

Minerals Affecting Female Fertility													
	Nr. of Studies	Ref.	Year	Study Type	Setting	*n*	Inclusion Criteria	Mean Age	Mean BMI	Minerals	Clinical Outcome	Serum/FF/ Whole Blood/ Supplement	Quality Rating
Embryo Development	3	[54]	2017	Prospective cohort study	Poland	221	IVF (ICSI)	25–35 years	22.18 + −3.28 kg/m^2^	Cd, Cu, Fe, Pb, Se, Zn	Higher levels of Cu, Zn, and Se in the follicular fluid	FF	Medium quality
		[55]	2023	Prospective, single-center, pilot study	USA	60	IVF (ICSI)	33.40 years	23.87 kg/m^2^	Cu, Zn, Li, Se, Mn, Mo, Cr, Fe	Cu and Mn positively influenced ovarian response and embryo development	Urine, plasma, and follicular fluid	Medium quality
		[27]	2022	Case-control study	China	231	IVF	20–40	22.43 ± 3.57 kg/m^2^	Cu, Zn, Se, and Co	Low Se levels in EEA case group and significant higher Co levels	Whole blood	Medium quality
Oocyte Quality	7	[53]	2006	Prospective cohort study	Massachusetts	18,555	Married, premenopausal women without a history of infertility	32.6 ± 3.6	Unknown	Fe	Iron supplement may decrease the risk of ovulatory infertility	Supplement	Medium quality
		[28]	2023	Case-control study	Austria	72	Women with unexplained infertility	18–40	24.0 (20.7; 27.4) (infertile), 21.2 (20.1; 22.3) (healthy)	Fe	Ferritin levels <30 µg/L associated with unexplained infertility	Serum	Medium quality
		[24]	2023	Observational study	Massachusetts	582	Women seeking infertility treatment	35	23.2 (21.2, 26)	Fe	Fe above 45 mg/day is associated with lower ovarian reserve	Questionnaire	Medium quality
		[21]	2020	Two-center cross-sectional study	Ghana	336	Nulliparous women	20–29	Unknown	Fe	Low Fe stores in nulliparous women	Serum	Low quality
		[18]	2015	Double-blinded randomized controlled trial	Egypt	200	IVF (ICSI)	29.53 ± 4.70 (calcium), 28.65 ± 4.91 (control)	26.46 ± 2.84 (calcium), 26.16 ± 2.63 (control)	Ca	Ca infusion reduce OHSS	Infusion	High quality
		[52]	2011	Prospective cohort	United Kingdom	30	IVF	32.7	25.4 (5.0) kg/m^2^	Hg, Zn, and Se	Zn and Se correlated positively with oocyte quality and with follicle number	Hair	Medium quality
		[51]	2023	Prospective study	Spain	93	ART	36.0 ± 3.9	24.7 ± 3.3 kg/m^2^	Al, B, Ba, Ca, Cd, Co, Cr, Cu, K, Li, Mg, Mn, Mo, Na, Ni, Pb, Sr, V, Zn	Fe positively influences oocyte quantity and maturity and high levels of Ca and Na negatively impact these outcomes.	FF	Medium quality
Oxidative Stress	2	[50]	2013	Prospective cohort	India	340	IVF	32.1	22.2 ± 2.67 (endometriosis), 21.6 ± 3.27 (tubal infertility)	Se, Zn, Cu, Fe, Pb, and Cd	Se reduces oxidative stress and improves oocyte and embryo quality. Increased levels of ROS, NO, LPO, Cd and Pb observed in women who did not become pregnant.	FF	Medium quality
		[56]	2016	Comparative study	Saudi	65	Recurrent miscarriage (RM)	19–24 years	Unknown	Zn, Cu, and Mn	Zn, Cu and Mn levels were significantly reduced in RM patients	Plasma and whole blood	Medium quality
Miscarriage	16	[49]	2023	Prospective cohort study design	China	200	IVF	32.33 ± 4.36 (live birth), 32.57 ± 3.84 (miscarriage)	23.06 ± 4.04 (live birth), 22.71 ± 3.55 (miscarriage)	V, Cu, Zn, Se and Mo	V, Cu, Zn and Se, were negatively correlated with miscarriage risk	Serum	Medium quality
		[29]	2015	Case-control	Poland	153	Miscarriage	30 years (miscarriage), 28 years (control)	21.10 kg/m^2^ (control), 22.43 kg/m^2^ (miscarriage)	Se, Zn Cu, Mn	Low level of Zn but high of Mn can affect the incidence of miscarriage	Serum	High quality
		[30]	2012	Case-control	Nigeria	69	Spontaneous abortion (SA)	30.09 ± 0.80 (control), 30.50 ± 0.88 (cases)	Unknown	Zn, Cu, Se, Fe, Mg, Mn, Cr, Pb, Cd	High Cd and Pb levels and low Zn and Cu contribute to recurrent SA	Serum	Medium quality
		[31]	2016	Case-control	Serbia	250	SA	27.46 ± 5.17 (healthy), 28.09 ± 6.34 (SA), 29.83 ± 6.17 (miscarriage)	Unknown	Cu	Significantly lower levels Cu and anti-oxidative enzymes in abortions	Serum	High quality
		[32]	2020	Case-control	China	150	SA	Unknown	Unknown	Pb	Pb level above 10 μg/L increases spontaneous abortion compared to levels below 5 μg/L	Whole blood	Medium quality
		[33]	2021	Case-control	Iran	206	SA	31.9 ± 6.0	26.2 ± 5.2	Pb, Ni, Sb	No statistically significant association between blood Pb levels and the risk of SA	Whole blood	Medium quality
		[33,34]	2013	Case-control	South Africa	48	RPL	32.5 (5.62) (miscarriage, 33 (5.87) (control)	31.9 (21.8) (miscarriage), 28.5 (6.3) (control)	Se	Low Se levels in case and control group	Hair	Medium quality
		[48]	2017	Prospective cohort	Poland	74	Autoimmune thyroid disease	30	24.3 ± 3.6 (case), 22.4 ± 2.9 (control)	Se	Se deficit during pregnancy	Serum	Low quality
		[47]	2011	Prospective cohort		1197	Preterm Birth	1.02 (0.94–1.10)	1.03 (0.97–1.08)	Se	Low serum Se at the end of the first trimester was related to preterm birth	Serum	Medium quality
		[35]	2013	Case-control	Indonesia	71	Miscarriage	28.72 ± 4.62 (control), 28.40 ± 4.10 (miscarriage)	Unknown	Se	Low serum Se levels are associated with miscarriage	Serum	Medium quality
		[25]	2002	Pilot Study	India	20	RPL	unknown	unknown	Se	Lower red cell Se levels in RPL group	Serum	Medium quality
		[36]	2019	Case-control	Russia	485	Woman with RPL and primary infertility	33.1 ± 5.0 (control), 33.4 ± 4.4 (pregnancy), 34.8 ± 6.3 (miscarriage), 35.5 ± 5.1 (primary infertility)	21.6 ± 2.2 (control), 22.3 ± 1.9 (pregnancy), 21.2 ± 2.0 (miscarriage), 21.0 ± 2.3 (primary infertility)	Cu, Fe, Mn	Low Cu levels were observed in those with miscarriage and infertility issues.	Serum	Medium quality
		[37]	2023	Case-control	Iran	120	SA	35–70 years	Unknown	Pb, As, Zn, and Se	Lower levels of Zn and Se and higher levels of Pb and As in SA	Whole blood	Medium quality
		[22]	2022	Cross-section	China	195	Miscarriage	30.87 ± 5.81 (control), 31.29 ± 5.07 (miscarriage)	21.80 ± 2.93 (control), 21.56 ± 2.44 (miscarriage)	Rb, Cu, Ba	High levels of Cu and Rb were associated with reduced risk for miscarriage	Whole blood	High quality
		[23]	2020	Cross-section	China	152	SA	30.0 (25.5–33.0) (non-pregnant), 30.0 (28.0–33.0) (pregnant), 32.0 (29.0–36.0) (SA)	20.4 (18.5–22.2) (non-pregnant), 20.2 (18.6–21.6) (Pregnant), 20.9 (19.9–22.9) (SA)	Mg, Cu, V, Sr, Sn, arsenic, antimony, and bismuth	Decreased levels of key pregnancy hormones (estradiol and progesterone) and higher levels of non-essential metals (like arsenic, antimony, and bismuth) and disrupted metabolism of essential metals (such as Mg, Cu and strontium) in SA	Serum	Medium quality
		[38]	2010	Case-control	Iran	351	SA	Unknown	Unknown	Pb	Low blood Pb levels (mean < 5 μg/dL) measured in early pregnancy may not be a risk factor for SA	Whole blood	Medium quality
Hormonal Regulation	1	[46]	2018	Prospective cohort	United States	259	Healthy women	18–44 years	>18 or <35 kg/m^2^	Mg, Mn; Na, P, Se, Zn, Ca, Cu, Fe, Mg, Mn, P, K, Se, Na, Zn	Low Na intake (<1500 mg) compared to higher intake (≥1500 mg) was associated with increased levels of FSH and LH hormones and decreased progesterone levels. Additionally, low intake of sodium and manganese was linked to an elevated risk of anovulation.	Serum	High quality
IVF Outcome	4	[45]	2021	Prospective cohort	China	305	IVF	31.4 ± 3.4	16.5–31 kg/m^2^	Zn	Lower Zn levels might be a risk factor for IVF-ET failure	Serum	High quality
		[19]	2022	Multicenter, randomized prospective study	Russia	110	IVF	20–35	18 ≤ BMI ≤ 30 kg/m^2^	Mg and Ca	Lower Ca/Mg ratio were associated with worse ART outcomes	Serum	High quality
		[44]	2017	Prospective cohort	New York		IVF	36 ± 4.9	23 ± 3.0	Mg and Ca	Increased estrogen levels and Mg and Ca levels during IVF stimulation	Serum	Medium quality
		[26]	2017	Pilot study	California	58	IVF	28–43	22.3 kg/m^2^	Co, Cr, Cu, Mo, Mn	Cr, Mn negatively impacted mature oocyte proportion, while FF Zn was inversely related to oocyte fertilization rates	FF	Medium quality
Environmental Exposure	1	[39]	2019	Case-control study	Japan	98	Infertile and healthy women	Unknown	Unknown	Hg and Se	Se and Hg molar ratio were significantly lower in the infertile group	Whole blood	Medium quality
Minerals as Biomarker	5	[20]	2011	Randomized controlled trial	Turkey	69	IVF	28.8 ± 3.2 years	Unknown	Se, Al, Cu, Zn, Mg	Lower Cu, Se, Zn in serum and follicular fluid decreased in women undergoing IVF	FF and serum	High quality
		[43]	2021	Prospective cohort	California	56	IVF	38.7	24.0 kg/m^2^	As, Hg, Cd, Pb, Cu, Mn, Se, and Zn	FF is a suitable source of biomarkers of As and Hg exposure in ovarian follicles	FF	Low quality
		[40]	2019	Case-control	China	195	IVF	34.28 (5.93) (case), 34.47 (3.73) (control)	Unknown	Cd. Cr, Se. As, Hg	Blood Cd >0.4 µg/L or urine Cr >2 µg/L might indicate an increased risk of SA	Whole blood and urine	Medium quality
		[41]	2018	Case-control	Poland	83	Miscarriage	29.90 ± 6.3 (case), 27.20 ± 3.5 (control)	22.89 ± 3.6 (case), 22.74 ± 3.2 (control)	Cd and Pb	High Cd and Pb concentrations in the blood and placenta in miscarriage	Whole blood and placenta tissue	Medium quality
		[42]	2023	Case-control	India	180	SA	28.19 ± 3.09 (control) 27.4 ± 4.4 (case)	24.32 ± 7.9 (control), 21.78 ± 2.8 (case)	Copper (Cu), Zinc (Zn), Magnesium (Mg)	Maternal serum Cu, Mg, Zn, and Iron (Fe) has the potential to forecast future occurrence of SA	Serum	Medium quality

**Table 3:** Recommended daily intake of minerals for female fertility.

Mineral	Recommended Daily Intake (Women, 19–50 Years)
Selenium	55–60 µg [63]
Zinc	8–11 mg [64]
Copper	0.9 mg [65]
Iron	18 mg [66]
Calcium	1000 mg [67]
Magnesium	310–320 mg [68]
Manganese	1.6–2 mg [69]

**Table 4:** Optimal serum levels of key minerals for supporting fertility.

Mineral	Optimal Serum Level
Selenium	8 µg/dL [63]
Zinc	80–120 µg/dL [64]
Copper	65–160 µg/dL [65]
Iron Ferritin	15–150 ng/mL [66]
Calcium	8.8–10.4 mg/dL [67]
Magnesium	1.8–2.3 mg/dL [68]
Manganese	4–15 µg/L [69]

**Table 5:** Clinical effects of mineral deficiencies and excesses.

Mineral	Effects of Deficiency	Effects of Excess
Selenium [63]	Increased oxidative stress, reduced fertility	Toxicity (e.g., hair loss)
Zinc [64]	Hormonal imbalance, higher miscarriage risk	Toxicity (e.g., gastrointestinal issues)
Copper [65]	Weakened immune function, increased miscarriage risk	Liver damage, oxidative stress
Iron [66]	Anemia, reduced ovarian reserve	Toxicity, gonadotoxicity
Calcium [67]	Bone weakness, increased miscarriage risk	Interference with absorption of other nutrients
Magnesium [68]	Higher preeclampsia rates, muscle cramps	Diarrhea, low blood pressure
Manganese [69]	Impaired ovulation, oxidative stress	Neurotoxic effects

## Data Availability

No new data were created or analyzed in this study.
